# IKKβ overexpression together with a lack of tumour suppressor genes causes ameloblastic odontomas in mice

**DOI:** 10.1038/s41368-019-0067-9

**Published:** 2020-01-02

**Authors:** Angustias Page, Ana Bravo, Cristian Suarez-Cabrera, Raquel Sanchez-Baltasar, Marta Oteo, Miguel Angel Morcillo, M. Llanos Casanova, Jose C. Segovia, Manuel Navarro, Angel Ramirez

**Affiliations:** 1 Molecular Oncology Unit. Centro de Investigaciones Energéticas, Medioambientales y Tecnológicas (CIEMAT), Madrid, Spain; 2 Instituto de Investigación 12 de Octubre i+ 12, Madrid, Spain; 3 Centro de Investigación Biomédica en Red de Cáncer (CIBERONC), Madrid, Spain; 4 Department of Anatomy, Animal Production and Veterinary Clinical Sciences, Laboratory of Pathology Phenotyping of Genetically Engineered Mice, Faculty of Veterinary Medicine, University of Santiago de Compostela, Lugo, Spain; 5 Biomedical Applications and Pharmacokinetics Unit, Centro de Investigaciones Energéticas, Medioambientales y Tecnológicas (CIEMAT), Madrid, Spain; 6 Hematopoietic Innovative Therapies Division. Centro de Investigaciones Energéticas, Medioambientales y Tecnológicas (CIEMAT). Centro de Investigación Biomédica en Red de Enfermedades Raras (CIBERER), Madrid, Spain; 7 Unidad Mixta de Terapias Avanzadas. Fundación Instituto de Investigaciones Sanitarias Fundación Jiménez Díaz, Madrid, Spain

**Keywords:** Oral cancer, Oral cancer, Cancer genetics

## Abstract

Odontogenic tumours are a heterogeneous group of lesions that develop in the oral cavity region and are characterized by the formation of tumoural structures that differentiate as teeth. Due to the diversity of their histopathological characteristics and clinical behaviour, the classification of these tumours is still under debate. Alterations in morphogenesis pathways such as the Hedgehog, MAPK and WNT/β-catenin pathways are implicated in the formation of odontogenic lesions, but the molecular bases of many of these lesions are still unknown. In this study, we used genetically modified mice to study the role of IKKβ (a fundamental regulator of NF-κB activity and many other proteins) in oral epithelial cells and odontogenic tissues. Transgenic mice overexpressing IKKβ in oral epithelial cells show a significant increase in immune cells in both the oral epithelia and oral submucosa. They also show changes in the expression of several proteins and miRNAs that are important for cancer development. Interestingly, we found that overactivity of IKKβ in oral epithelia and odontogenic tissues, in conjunction with the loss of tumour suppressor proteins (p53, or p16 and p19), leads to the appearance of odontogenic tumours that can be classified as ameloblastic odontomas, sometimes accompanied by foci of secondary ameloblastic carcinomas. These tumours show NF-κB activation and increased β-catenin activity. These findings may help to elucidate the molecular determinants of odontogenic tumourigenesis and the role of IKKβ in the homoeostasis and tumoural transformation of oral and odontogenic epithelia.

## Introduction

The IKK complex is responsible for the regulation of the NF-κB family of transcription factors, which regulates genes associated with cell survival and increased proliferation. Deregulated NF-κB activation underlies disease states in many organs, including chronic inflammation and cancer. The IKK complex is formed by two catalytic subunits with kinase activity (IKKα and IKKβ) and one regulatory subunit (IKKγ or NEMO).^1^ In addition to the role of IKKβ as a positive regulator of NF-κB activity, it interacts with, phosphorylates, and thereby modifies the activity of a plethora of proteins implicated in a number of functions.^1,2^ Furthermore, it has recently been reported that the IKK complex acts as a general regulator of gene expression by modifying mRNA stability.^3^ Thus, IKKβ is able to regulate cellular physiology in different ways and, not surprisingly, changes in the activity of IKKβ are associated with cancer in several cell types. Interestingly, IKKβ can either promote or prevent tumour development, depending on the cell type and other circumstances that are not yet well understood, probably due to the large number of proteins regulated by this kinase.^4^

Previously, we generated transgenic mice overexpressing IKKβ in basal cells of the stratified epithelia and in exocrine glands. In addition to other phenotypes, IKKβ overexpression in these mice led to greater numbers of CD45^+^ haematopoietic cells as well as granulocytes (Gr-1^+^), macrophages (F4/80^+^) and B cells (B220^+^) in the forestomach epithelium^5^ and to the development of supernumerary teeth due to reduced apoptosis and upregulation of the WNT signalling pathway in the embryonic incisor bud epithelium of K5-IKKβ mice.^6^ Carcinogenesis experiments performed in a genetic background prone to tumour development through the expression of an active form of RAS revealed that K5-IKKβ mice are resistant to skin cancer,^7^ but they develop more malignant tumours than control littermates in the forestomach and the palate.^5^ In the course of these experiments, we crossed the K5-IKKβ transgene into backgrounds lacking p53 in epithelial cells (p53^EKO^/K5-IKKβ mice) or lacking p16 and p19 in every cell (*Ink4a/Arf* ^KO^/K5-IKKβ). Surprisingly, a high percentage of these mice developed spontaneous odontogenic tumours.

Odontogenic tumours are a heterogeneous group of lesions of the oral cavity that result in the formation of tumoural structures that differentiate as teeth. Their classification is still under debate and has been recently modified by the World Health Organization.^8^ These lesions are currently classified as odontogenic cysts and odontogenic tumours (benign or malignant), and each of these categories is in turn classified into a number of subcategories. Although the aetiology of these tumours is unknown in many cases, activating mutations in the Hedgehog (mutations in *SMO* and *PTCH1*) and mitogen-activated protein kinase (MAPK; mutations in *BRAF*, *RAS, PIK3CA* or *FGFR2*) pathways have been identified in benign and malignant odontogenic tumours. Other genetic alterations found in odontogenic tumours are associated with the WNT pathway (in *APC*, *MCC* and *CTNNB1*) and other genes that are commonly mutated in cancer (i.e., *CDKN2A* or *TP53*).^8^ In addition, epigenetic alterations have been described in these tumours.^9^

Transgenic mice in which WNT signalling is activated through the expression of stabilized forms of β-catenin in oral epithelia resulted in the development of supernumerary teeth.^10,11^ Although these animal models do not survive postnatally, precluding the study of tumour formation in adulthood, the alterations that they develop resemble odontogenic tumours. Postnatal activation of the WNT pathway in dental stem cells results in odontoma formation.^12^ However, other determinants of odontogenic tumours are still unknown, and there is a need for additional animal models to reveal the genetic diversity underlying odontogenic tumours.

Here, we analyse the effect of IKKβ overexpression in epithelial cells of the gingiva and palate and the inflammatory disease caused by IKKβ in the oral epithelia as well as the associated changes in the expression of several key proteins and miRNAs that are important for cancer development. We demonstrate that the overactivity of IKKβ in oral epithelial cells and odontogenic tissues, in conjunction with the loss of tumour suppressor proteins (p53, or p16 and p19), leads to the appearance of odontogenic tumours. Molecular characterization of these tumours indicates that they show NF-κB activation and increased β-catenin activity. These findings shed some light on the role of IKKβ in the homoeostasis and tumoural transformation of oral and odontogenic epithelia.

## Results

### Chronic inflammation of oral epithelia in K5-IKKβ transgenic mice

**Fig. 1:**
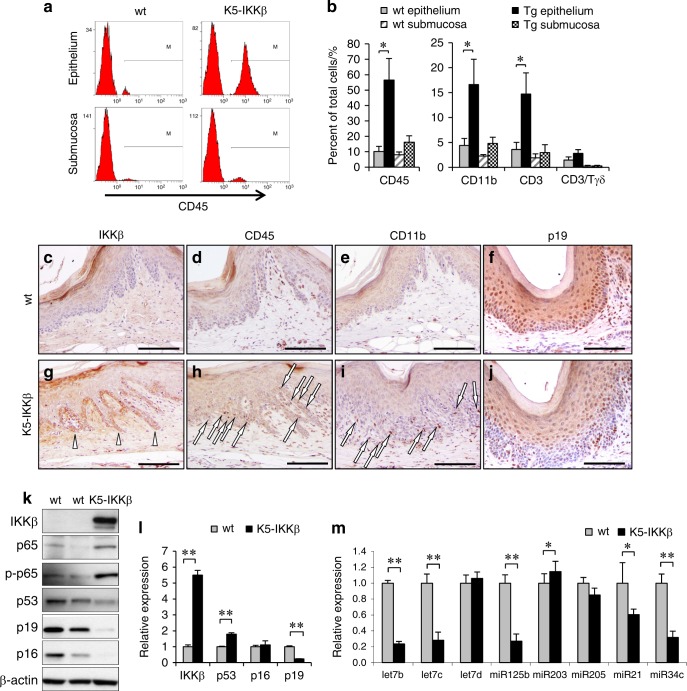
IKKβ leads to changes in immune cell populations and the expression of relevant proteins and miRNAs in cancer in oral epithelia. **a** Examples of flow cytometry plots showing the increase in CD45^+^ cells in both the oral epithelium and oral submucosa in K5-IKKβ transgenic mice compared to wt littermates. **b** Frequency of CD45, CD11b, CD3 and γδT cells in the oral epithelium and submucosa of animals of the indicated genotypes. **c**–**j** Immunohistochemical staining for IKKβ, CD45, CD11b and p19 in palate sections in wt (**c**–**f**) and K5-IKKβ mice (**g**–**j**). **k** Western blots for the indicated proteins in protein extracts from cultured oral keratinocytes. **l** RT-qPCR analysis of mRNAs for tumour suppressor proteins. **m** RT-qPCR analysis of the indicated miRNAs in oral keratinocytes of the indicated genotypes. *P < 0.05; **P < 0.01. Bar: 100 μm.

Previously, we reported that K5-IKKβ transgenic mice develop histologic and inflammatory alterations in both the palate and the non-glandular stomach, which could underlie their predisposition to develop tumours in the oral epithelia.^5^ With the aim of understanding the role played by IKKβ in the oral epithelia, we studied these organs in K5-IKKβ mice in depth. In in vitro studies with cultured oral keratinocytes, K5-IKKβ cells showed more proliferation (measured as BrdU incorporation) and fewer senescent cells (measured as senescence-associated β-gal activity) than cultured wild-type (wt) oral keratinocytes (Supplementary Figs. 1, 2); in addition, K5-IKKβ oral keratinocytes formed a greater number of colonies (though smaller in size) when seeded at low density (Supplementary Fig. 3), although they were not fully transformed, as they were not able to grow when injected subcutaneously into nude mice (not shown). Next, we studied the infiltrating populations in both the epithelium and the submucosa of the palate by flow cytometry (Fig. 1a, b). Interestingly, approximately 10% of the cells from the epithelia of wt mice and 6.6% from the submucosa were not of epithelial origin but were infiltrating haematopoietic (CD45^+^) cells. Almost half of these cells could be classified as either macrophages or T-lymphocytes based on the labelling obtained with CD11b (also known as Mac-1) and CD3 antibodies, and some of these T-cells were of the γδ subtype, similar to the dendritic epidermal T cells residing in the skin. Interestingly, epithelial IKKβ overexpression in transgenic (tg) mice led to a large (statistically significant) increase in haematopoietic CD45^+^ cells in the palate epithelia, where haematopoietic infiltrating cells represented more than half of the total cell number (Fig. 1b). The increases in epithelial macrophages and oral dendritic cells were also noticeable and statistically significant in the palate epithelium of tg mice in comparison to wt. Additionally, we found a modest but statistically non-significant increase in these cell types in the oral submucosa (Fig. 1b). Immunohistochemical staining of palate sections verified the overexpression of IKKβ (Fig. 1c, g; arrowheads) and the presence of more CD45^+^ and CD11b^+^ cells in transgenic samples (Fig. 1d, e, h, i; arrows).

### Alterations in the expression of tumour suppressor proteins and microRNAs in K5-IKKβ oral keratinocytes

We next studied the expression levels of several genes and microRNAs (miRNAs) that are important for tumour development in cultured oral epithelial cells from wt and tg mice with IKKβ overexpression. K5-IKKβ oral keratinocytes showed increased phosphorylation of Ser^536^ of p65, indicative of NF-κB activation (Fig. 1k). Interestingly, in contrast to the increased expression of p16, p19 and p53 found in murine skin keratinocytes overexpressing IKKβ,^7^ oral keratinocytes overexpressing IKKβ expressed lower levels of these tumour suppressor proteins (Fig. 1k), suggesting that the pathways modified by IKKβ in oral and skin keratinocytes are at least partially different. Reduced expression was confirmed at the mRNA level for p19, but not for p16 and p53, suggesting the involvement of some mechanism of posttranscriptional regulation (in fact, we observed an increase in the level of p53 mRNA; Fig. 1l). Immunohistochemical studies confirmed the lower expression of p19 in K5-IKKβ oral epithelial cells in vivo, where most of the nuclei of the basal layers were negative for p19 staining (Fig. 1f, j). In summary, we have found that overexpression of IKKβ in oral epithelial cells leads to decreased expression of several tumour suppressor proteins.

To characterize the expression of other important players in tumoural transformation, we studied the expression of several microRNAs previously described to be altered in cancer (Fig. 1m). K5-IKKβ oral keratinocytes expressed substantially lower amounts of several members of the let-7 group, miR-125b, miR-21 and miR-34c than wt oral keratinocytes. Interestingly, let-7 family members, miR-125b and miR-34 tend to be downregulated in tumoural samples, including those from the oral cavity.^13–16^ In summary, K5-IKKβ oral epithelial cells presented lower levels of a number of miRNAs that are usually downregulated in transformed states. In contrast, we did not observe a decrease in miR-203, which is another microRNA whose downregulation is usually associated with tumoural transformation, nor a significant difference for miR-205 (Fig. 1m).

### Loss of tumour suppressor proteins led to spontaneous odontogenic tumours in K5-IKKβ transgenic mice

**Fig. 2:**
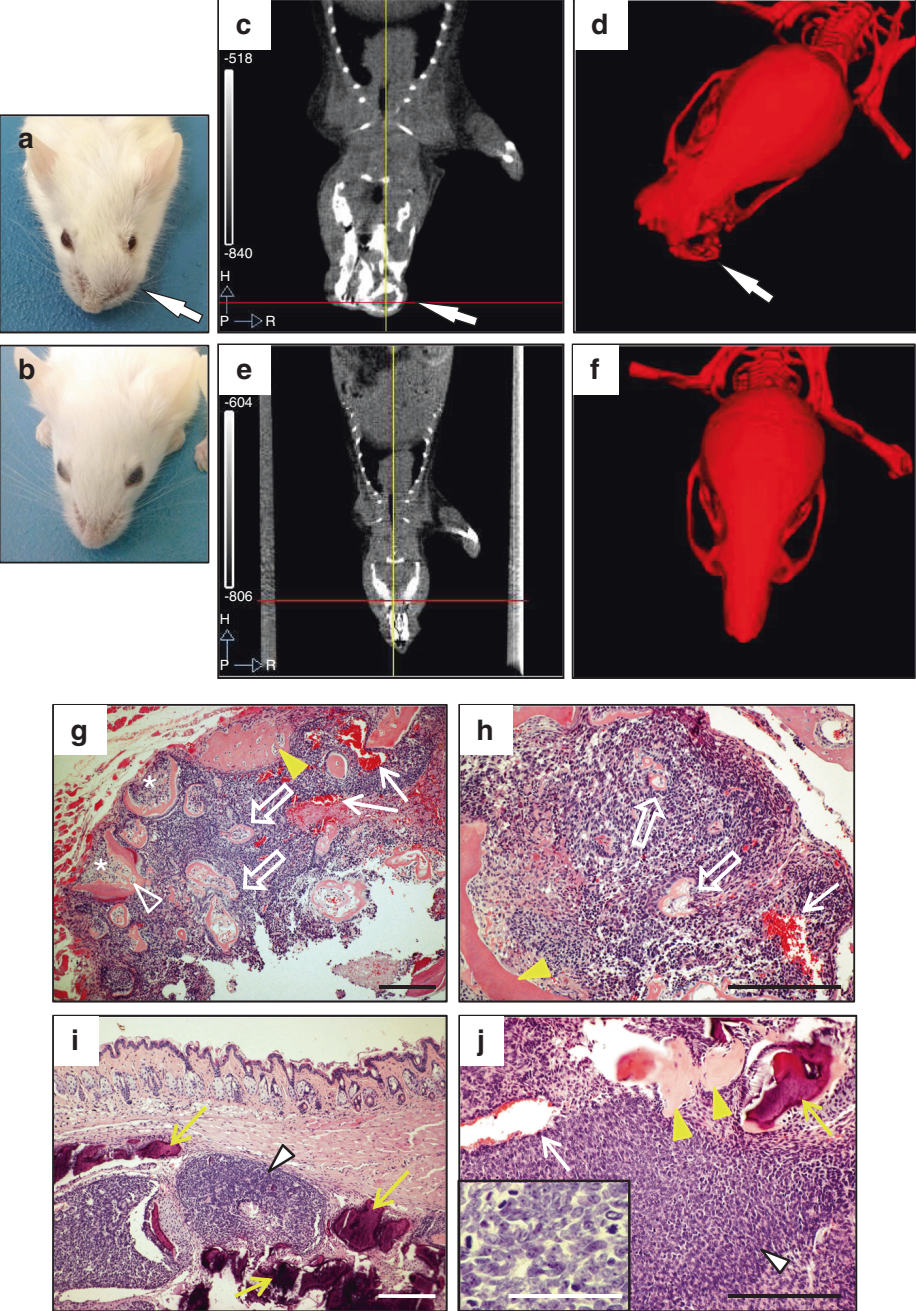
Odontogenic tumours in K5-IKKβ mice lacking either p53 or the p16 and p19 tumour suppressor proteins. **a**–**f** Macroscopic and computed tomography (CT) images of an Ink4a/Arf KO/K5-IKKβ mouse with an odontogenic tumour (**a**, **c**, **d**) and a wild-type mouse (**b**, **e**, **f**). **a**, **b** External appearance of an Ink4a/Arf KO/K5-IKKβ mouse affected by an odontogenic tumour arising in the left maxilla (**a**) and a wild-type mouse (**b**). **c**, **e** CT images of the heads of these mice. **d**, **f** Reconstructed 3D CT scan images of the skulls of these mice. The arrows indicate a left maxilla deformity caused by the odontogenic tumour. **g**–**j** HE staining of histological sections from ameloblastic odontomas in p53EKO/ K5-IKKβ (**g**, **h**) and Ink4a/Arf KO/K5-IKKβ (**i**, **j**) mice. The tumours showed two components: hard tissues producing primitive denticles (bold arrows in **g**, **h**) or mature tooth buds (arrowhead in **g**) containing stellate reticulum-like cells (asterisks in **g**) and soft tissues composed of proliferating ameloblasts; these areas tended to form foci of secondary ameloblastic carcinoma (white arrowheads in **i**, **j**) that infiltrated and destroyed the alveolar bone (yellow arrows in **i**, **j**). These malignant foci showed highly proliferative and hyperchromatic ameloblastic cells that displayed severe pleomorphism and abundant mitosis, some of which were aberrant (inset in **j**). The tumours commonly showed multifocal haemorrhages (white arrows in **g**, **h**, **j**) and new bone formation (yellow arrowheads in **g**, **h**, **j**). Compare the poorly calcified eosinophilic new bone matrix (yellow arrowheads in **g**, **h**, **j**) with the mature mineralized basophilic alveolar bone (yellow arrows in **i**, **j**). Bar: 100 μm, except in the inset in **j** (33 μm).

In the context of our study on the role of IKKβ in skin cancer, we generated cohorts of K5-IKKβ transgenic mice that also lacked either p53 in stratified epithelial tissues (p53^EKO^/K5-IKKβ mice) or p16 and p19 in every cell (*Ink4a/Arf* ^*KO*^/K5-IKKβ mice). Interestingly, in addition to an *Ink4a/Arf-*dependent antitumoural role of IKKβ in skin cancer,^7^ we observed that overexpression of IKKβ in the absence of either epithelial p53 or both p16 and p19 led to the appearance of maxillary tumours (usually monolateral) that caused facial distortion and swelling in some mice (Fig. 2a, b). These tumours appeared as radiopaque protuberant masses that expanded past the natural snout line and were associated with bone loss (Fig. 2c, d). We did not observe any odontogenic tumours in mice lacking the K5-IKKβ transgene, independently of the p53 or p16/p19 genetic background, or in K5-IKKβ mice bearing wt copies of p53 and *Ink4a/Arf* (Fig. 2e, f). By contrast, p53^EKO^/K5-IKKβ and *Ink4a/Arf* ^*KO*^/K5-IKKβ mice developed similar odontogenic tumours at frequencies of 13.6% and 34.4%, respectively (Table 1). These differences in the distribution of odontogenic tumours in mice bearing and lacking the K5-IKKβ transgene were statistically significant (*P* < 0.0001, Fisher exact test). We did not observe differences between the sexes in the incidence of odontogenic tumours in either p53^EKO^/K5-IKKβ or *Ink4a/Arf* ^*KO*^/K5-IKKβ mice.

**Table 1:** Spontaneous ameloblastic odontomas and other epithelial tumours in p53^EKO^/ K5-IKKβ, p53^EKO^, *Ink4a/Arf* ^KO^/K5-IKKβ, and *Ink4a/Arf* ^KO^ mice.

Items	p53^EKO^/K5-IKKβ	p53^EKO^	*Ink4a/Arf*^KO^/ K5-IKKβ	*Ink4a/Arf*^KO^
Mice analysed	59	55	61	43
Mice with tumours	26	31	28	9
Number of epithelial tumours	35	46	28	2
Mice with ameloblastic odontomas	8	0	21	0
Number of ameloblastic odontomas	10	0	21	0
Ameloblastic odontomas vs. epithelial tumours/%	28.6	0	75.0	0
Mice with ameloblastic odontomas/%	13.6	0	34.4	0
Age of odontogenic tumour appearance/months	5–12	–	6–10	–

Histological examination of these tumours revealed characteristics of ameloblastic odontomas (or odontoameloblastomas) that agreed with the international classification of dental tumours in mice.^17^ The ameloblastic odontomas of both p53^EKO^/K5-IKKβ and *Ink4a/Arf*^*KO*^/K5-IKKβ mice appeared as well-circumscribed tumours that showed differentiation into hard dental tissues, normally located in the central areas of the tumour, and soft tissues composed of proliferating ameloblastic epithelium, generally located at the periphery of the tumour (Fig. 2g–j). The hard tissues of the tumours showed different degrees of morphologic differentiation of tooth structures, ranging from primitive tooth buds (denticles; open arrows in Fig. 2g, h) to mature teeth with distinct ameloblasts, odontoblasts, dentin-like and enamel-like material and cementum (Fig. 2g, open arrowhead). The inner areas of these tooth structures usually contained cells that resembled the stellate reticulum (Fig. 2g, asterisks). It was not rare to find multifocal areas of haemorrhage (Fig. 2g, h, j white arrows), necrosis, inflammation and poorly calcified new bone formation (Fig. 2g, h, j, yellow arrowheads). Although ameloblastic odontomas usually do not metastasize,^17^ the soft ameloblastic epithelium of the periphery of the tumours that developed in both p53^EKO^/K5-IKKβ and *Ink4a/Arf*^*KO*^/K5-IKKβ mice tended to form foci of highly proliferative and hyperchromatic solid cellular masses (Fig. 2i, j, white arrowheads) showing severe pleomorphism and abundant mitosis, suggestive of the development of secondary ameloblastic carcinoma (Fig. 2j, inset); these foci displayed local infiltration and destruction of the alveolar bone (Fig. 2i, j, yellow arrows) and subcutaneous tissues, even leading to infiltration and ulceration of the skin.

### Odontogenic tumours derived from IKKβ-expressing cells showed activation of proliferative pathways

**Fig. 3:**
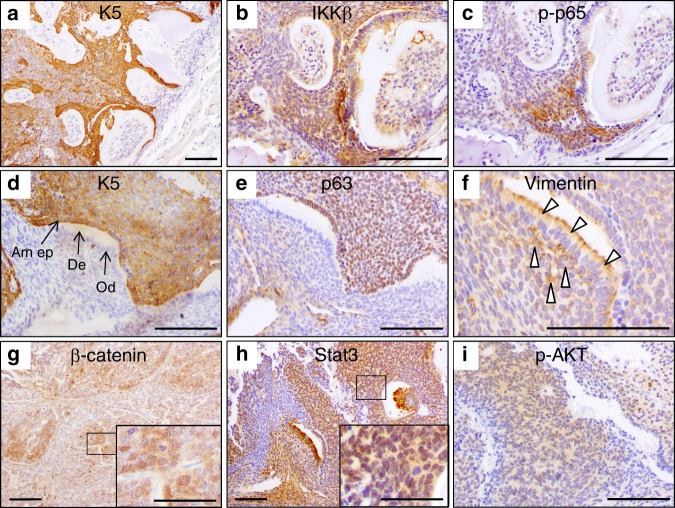
Immunohistochemical analyses of ameloblastic odontomas. **a**–**i** Immunohistochemical staining for keratin K5 (**a**, **d**), IKKβ (**b**), phospho-p65 (**c**), p63 (**e**), vimentin (**f**), β-catenin (**g**), STAT3 (**h**) and phospho-AKT (**i**) are shown. Am ep, ameloblastic epithelia; De, dentin; Od, odontoblasts. Bar: 100 μm, except in the insets in **g** and **h** (50 μm).

We next immunohistochemically studied the expression of epidermal and mesenchymal markers in odontogenic tumours as well as the expression of proteins that are important in the transduction of prosurvival and proliferation signals that are frequently activated in cancer. The ameloblastic odontomas of mice overexpressing IKKβ maintained the epithelial differentiation of the soft ameloblastic epithelium, as it was mainly positive for staining with an antibody specific for keratin K5 (Fig. 3a). The ameloblastic regions of the tumours that expressed keratin K5 at higher levels also showed detectable expression of IKKβ (Fig. 3b) and increased activation of NF-κB, measured as staining for phospo-p65 (Fig. 3c). Notably, the hard and internal portions of the denticles (dentin, odontoblasts and stellate reticular cells) did not express keratin K5 (Fig. 3a, d) or p63 (Fig. 3e) but were positive for vimentin staining (Fig. 3f, arrowheads), indicating the mesenchymal differentiation of these cells, as occurs in normal teeth. The ameloblastic odontomas showed activation of the canonical WNT/β-catenin, STAT3 and AKT signalling pathways in the ameloblastic epithelium, as they exhibited nuclear β-catenin and STAT3 staining, especially in the foci of secondary ameloblastic carcinoma (insets in Fig. 3g, h), which were also positive for staining with an antibody specific for phospho-AKT (Ser473), an active form of AKT (Fig. 3i).

**Fig. 4:**
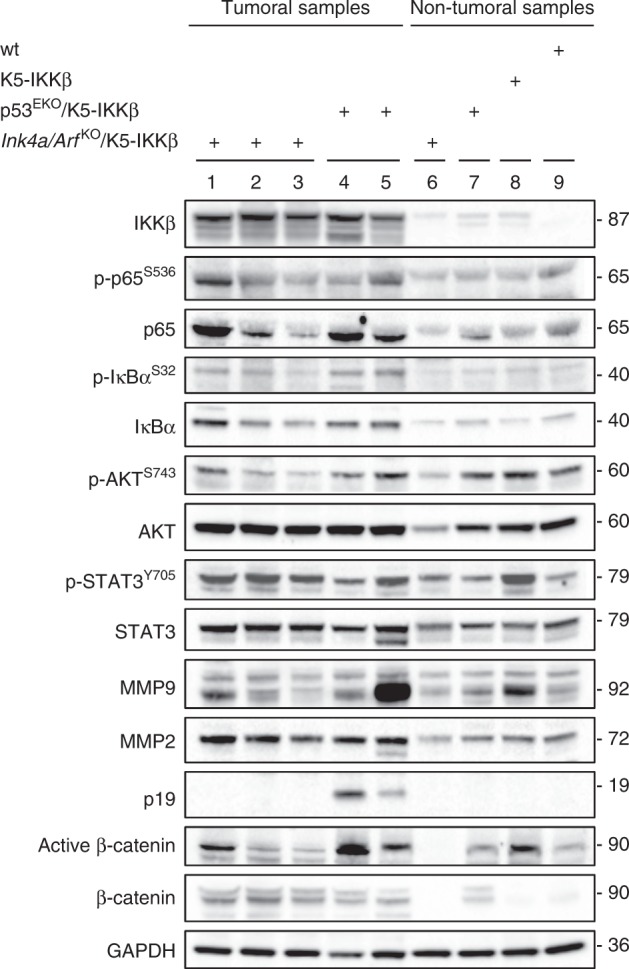
Western blot analysis of odontogenic tumours and non-tumoural tissues from normal maxillae of the indicated genotypes. Odontogenic tumours (lanes 1–5) showed increased expression of IKKβ, increased NFκB activation and increased expression or activity of the AKT, STAT3 and WNT/β-catenin pathways as well as increased expression of MMP2 compared to non-tumoural oral and odontogenic tissues (lanes 6–9).

These results were confirmed by western blot analysis of protein extracts from several odontogenic tumours. We analysed three ameloblastic odontomas from *Ink4a/Arf* ^*KO*^/K5-IKKβ and two from the p53^EKO^/K5-IKKβ background as well as non-tumoural oral and odontogenic tissues from the genotypes indicated in Fig. 4. All the tumours (lanes 1–5) expressed higher levels of IKKβ than the non-tumoural oral and dental tissues of K5-IKKβ (lanes 6–8) or wild-type mice (lane 9). In addition, tumoural samples showed increased levels of phospho-p65 and phospho-IκBα, indicative of NF-κB activation, and increased expression (and, to a lesser extent, phosphorylation) of AKT and STAT3 than non-tumoural samples. Odontogenic tumours showed increased total and active β-catenin as well. Interestingly, the tumoural samples also expressed more MMP2; as MMP2 degrades type IV collagen, the most abundant component of the basement membrane, its overexpression could contribute to the aggressive local behaviour and metastatic potential (see below) observed in the foci of secondary ameloblastic carcinoma that developed in the pre-existing ameloblastic odontomas. Notably, we only detected p19 in tumoural samples from animals with a wild-type *Ink4a/Arf* locus (lanes 4–5).

In summary, the analyses of odontogenic tumours and non-tumoural samples by immunohistochemistry and western blotting indicated activation of the NF-κB, AKT, STAT3 and WNT/β-catenin pathways in tumoural samples, independent of their genetic background.

### Odontogenic tumours in K5-IKKβ transgenic mice can metastasize

**Fig. 5:**
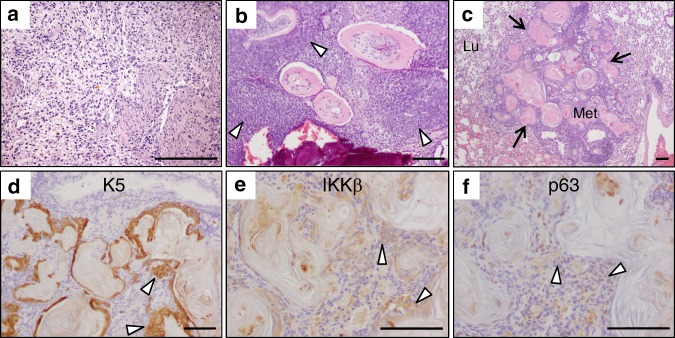
Metastasis from odontogenic tumours. **a** Metastasis observed in a cervical lymph node showing poorly differentiated ameloblasts and stellate reticulum-like cells. **b**, **c** primary ameloblastic odontoma with foci of ameloblastic carcinoma (arrowheads in **b**) and microscopic metastasis of the lung showing ameloblast proliferation with abundant foci of squamous metaplasia (arrows in **c**) in an Ink4a/Arf KO/K5-IKKβ mouse. **d**–**f** Immunohistochemical staining of the same lung metastasis shown in c with the indicated antibodies; note that only proliferating ameloblasts showed specific staining, while the foci of squamous metaplasia appeared unstained. Lu, lung tissue; Met, lung metastasis. Bar: 100 μm.

Ameloblastic odontomas are considered benign or low-grade malignant neoplasias that usually do not metastasize but behave as locally aggressive growths invading and destroying surrounding tissues, including bone.^17^ By contrast, the tumoural lesions observed in K5-IKKβ mice simultaneously lacking p53 or p16 and p19 were able to metastasize (Fig. 5). We observed metastasis to a cervical lymph node, which was filled with cells similar to stellate reticulum cells (Fig. 5a) in an *Ink4a/Arf*^*KO*^/K5-IKKβ mouse. In another *Ink4a/Arf*^*KO*^/K5-IKKβ mouse, we found an ameloblastic odontoma with foci of secondary ameloblastic carcinoma around the denticles (Fig. 5b, arrowheads), which developed microscopic metastatic foci in the lung, showing a cellular pattern similar to secondary ameloblastic carcinoma with abundant areas of squamous differentiation (Fig. 5c, arrows). The metastatic nature of this lesion was confirmed by its positive immunohistochemical staining for keratin K5, IKKβ and p63 (arrowheads in Fig. 5d–f). From these results, we conclude that the ameloblastic odontomas developed as a consequence of IKKβ overexpression in the context of a lack of tumour suppressor proteins are able to evolve into secondary ameloblastic carcinomas and metastasize to regional lymph nodes and the lung.

## Discussion

IKKβ exerts its multiple functions by positively regulating the activity of NF-κB but also via the phosphorylation and, hence, regulation of a number of other substrates that are important in multiple pathways affecting cell physiology.^1,2^ The role of IKKβ in cancer is consequently complex, showing both tumour-promoting and tumour-repressing activities, depending on the tissue and the cellular context.^4^

NF-κB drives a proinflammatory pathway that is constitutively activated and promotes invasion in oral squamous cell carcinomas (SCC).^18^ In this report, we have characterized the frequency of infiltrating haematopoietic cells in the oral epithelia of both wild-type and K5*-*IKKβ transgenic mice. The presence of immune cells in the oral mucosa is not surprising, as the oral cavity represents a gate for the entry of microorganisms into the body. Notably, approximately 10% of the cells in the oral epithelia of wild-type mice are of immune origin (i.e., CD45^+^ cells). In addition, IKKβ overexpression triggers a strong oral inflammatory response, as the percentage of immune cells increases up to more than 50% of the total cells in the oral epithelia. Accordingly, in the underlying submucosa, there is also a marked inflammatory response, although it is not as strong as in the oral epithelium. Some ameloblastic carcinomas in humans have been proposed to develop from ameloblastomas.^19^ Our results suggest that increased IKKβ expression provokes NF-κB activation and a strong inflammatory response that could contribute to the tumoural transformation of the odontogenic epithelium through the uncontrolled release of inflammatory cytokines and facilitate the development of a secondary ameloblastic carcinoma in a pre-existing ameloblastic odontoma, although the existence of other driving factors cannot be ruled out.

miRNAs are small non-coding RNAs that regulate gene expression by modifying the stability and transcription rate of target messenger RNAs. Each miRNA usually regulates a number of different targets and, hence, shows pleiotropic effects. Not surprisingly, miRNA deregulation is an important player in cancer pathogenesis, including oral SCC and head and neck cancer.^15^ The miRNAs that were downregulated by IKKβ overexpression included let-7b, let-7c, miR-125b, miR-21 and miR-34c; interestingly, all of these miRNAs are functionally related to tumoural transformation.

miRNAs of the let-7 family promote cellular differentiation and are considered tumour suppressors. These miRNAs are frequently expressed at low levels in human cancers and cancer stem cells. Among their targets, there are several cancer genes, such as *RAS, MYC, HMGA2* and *STAT3*,^20,21^ and a negative feed-back loop between let-7 members and β-catenin has been described.^22^ Therefore, the low levels observed for let-7b and let-7c could be important in the development of ameloblastic odontomas and secondary ameloblastic carcinomas in K5-IKKβ mice or, alternatively, could be secondary to β-catenin overactivity.

Although the function of the miR-125 family is somewhat controversial, its members are known to target several oncogenes, transcription factors and growth factors, and its function is tumour suppressive in a number of organs and tissues, including ectodermal derivatives.^23^ miR-125b is downregulated in oral SCC, and low expression of miR-125b contributes to head and neck cancer development;^14^ moreover, the restoration of miR-125b expression in oral SCC cell lines reduces the proliferation of these cells.^13^

miR-34c belongs to a family of microRNAs that are induced by p53 and cause cell cycle arrest and apoptosis, partially by targeting the expression of c-MYC.^24^ This miRNA also regulates multiple components of the WNT pathway, including β-catenin, and acts as a tumour suppressor miRNA in many cell types.^25^ Restoration of miR-34c expression leads to a decrease in the migration, invasion and metastatic potential of nasopharyngeal carcinoma cell lines.^26^ Notably, miR-34c is expressed in oral keratinocytes at much higher levels than in skin keratinocytes (more than 150 times; results not shown). This difference indicates that the functions exerted by this specific microRNA could be more important in oral epithelia than in skin. More studies are needed to determine whether the different effects of IKKβ on tumoural transformation in oral and skin keratinocytes are mediated by miR-34c.

The reduced level of miR-21 found in K5-IKKβ oral keratinocytes is surprising, as this miRNA is considered to be oncogenic and is overexpressed in many types of tumours. miR-203 is differentially expressed in the epithelium and mesenchyme of dental primordia, and hence, it could be important in tooth organogenesis.^27^ Thus, the overexpression of miR-203 found in K5-IKKβ oral keratinocytes could contribute to the development of ameloblastic odontomas and secondary ameloblastic carcinomas observed in p53^EKO^/K5-IKKβ and *Ink4a/Arf*^KO^/K5-IKKβ mice.

Under treatment with oral carcinogens, IKKβ promotes the appearance of more oral tumoural lesions (and more malignant lesions) in the palate and forestomach in a genetic background prone to the development of epithelial cancers (TgAC mice).^5^ Interestingly, these mice do not develop odontogenic tumours. By contrast, the cooperation of IKKβ overexpression with the lack of tumour suppressor proteins (p53 or p16 and p19) leads to the appearance of spontaneous ameloblastic odontomas in ~25% of animals, indicating that although the overexpression of IKKβ makes odontogenic epithelia prone to tumoural transformation, a concomitant lack of tumour suppressor proteins is needed for odontogenic tumour formation.

There are no published data regarding the genomic alterations present in ameloblastic tumours in repositories of genomic data for human cancers such as TCGA. Nevertheless, data available at cBioportal indicate that the most mutated genes in head and neck SCC include *TP53* (71.5%) and *CDKN2A* (the human locus that encodes p16 and p14, which is the human equivalent of murine p19; 22.1%). The implication of *CDKN2A* in the pathogenesis of odontogenic and other head and neck cancers is reinforced by the characterization of *CDKN2A* as a susceptibility locus for nasopharyngeal carcinoma in a genome-wide association study performed in a Chinese population;^28^ in addition, it has been suggested that the methylation of the *CDKN2A* locus is an important mechanism of odontogenic tumourigenesis,^29,30^ and loss of heterozygosity is observed for both *TP53* and 9p22-p21 (the genomic region where the *CDKN2A* locus occurs) in odontogenic tumours.^31^

At present, it is uncertain which of the proteins encoded by the *CDKN2A* locus needs to be lost to cooperate with IKKβ overexpression in odontogenic tumour formation. Mice lacking both p16 and p19 develop tumours, mainly sarcomas and lymphomas, but not odontogenic tumours. Mice that are null for p19 (but not for p16), generated by deleting exon E1β,^32^ develop tumours similar to those observed in p16 and p19 double-null mice, although a lack of p19 in association with Tax oncogene expression has been implicated in osteosarcoma development.^33^ Wild-type keratinocytes of the oral epithelia express p16 at higher levels than skin keratinocytes, suggesting that p16 loss could be important in oral tumourigenesis. Nevertheless, the deletion of either p16 or p19 individually in an animal model overexpressing IKKβ would allow the specific role of these proteins in the development of odontogenic tumours to be discerned.

β-catenin deserves particular attention as a possible driver of odontoma formation, as WNT overactivity causes the development of supernumerary teeth,^10,11^ and mice with increased WNT activity in the oral epithelium develop odontomas.^12^ In addition, β-catenin expression appears to increase with the aggressiveness of odontogenic lesions, which also increases its nuclear localization.^34^ Accordingly, through western blotting and immunohistochemical analyses, we detected increased β-catenin levels and/or activation in the ameloblastic odontomas of our animal models, especially in the areas of secondary ameloblastic carcinoma. Therefore, p53^EKO^/K5-IKKβ mice and *Ink4a/Arf*^*KO*^/K5-IKKβ mice represent good models for studying the pathogenesis of the progression to secondary ameloblastic carcinoma from pre-existing ameloblastic odontoma and are also very useful for testing the efficacy of IKKβ or WNT/β-catenin inhibitors in preventing odontogenic tumour development.

In summary, our results show that IKKβ overactivity in oral and odontogenic epithelia leads to changes in both the cellular composition of the epithelia and the expression of several tumour suppressor proteins and miRNAs that result in a preneoplastic state of the cells. IKKβ overactivity in transgenic mice in conjunction with the absence of tumour suppressor proteins (such as p53, p16 and/or p19) leads to the appearance of odontogenic tumours, which show activation of the WNT/β-catenin pathway. Therefore, these results indicate the possibility of treating ameloblastic odontomas with pharmacologic inhibitors of IKKβ or WNT/β-catenin to prevent their evolution to ameloblastic carcinoma.

## Materials and methods

### Mice and genotyping

All mouse husbandry and experimental procedures were performed according to European and Spanish laws and regulations and were approved by the local Animal Ethical Committee and the competent authority (code PROEX 086/15).

The genotyping of K5-IKKβ, p53^EKO^/K5-IKKβ and *Ink4a/Arf* ^KO^/K5-IKKβ mice was performed by PCR analysis of tail DNA as previously described.^35–38^ K5-IKKβ mice are available from the European Mouse Mutant Archive (code EM:09179). The expected genotypes were obtained in roughly Mendelian rates in the different crosses.

### Histology and immunohistochemistry

Mouse tissues were dissected, immediately fixed in 10% buffered formalin or 70% ethanol, and embedded in paraffin. Five micron thick sections were used for H&E staining or immunohistochemical preparations. The antibodies used are listed in Supplementary Table S1. Secondary antibodies were purchased from the Jackson Immunoresearch Laboratory. Immunoreactivity was revealed using an ABC-peroxidase system (Vector Laboratories), and the sections were lightly counterstained with haematoxylin. Control experiments omitting the primary antibody produced no signal.

### Culture of oral keratinocytes

Epithelial and submucosal cells were harvested from the oral tissue (inner cheek and palate) of 2-day-old K5-IKKβ and control mice as described previously^39^ with minor modifications. The samples were incubated overnight at 4 °C with 1 unit per mL dispase (Sigma, P-3417); then, the oral epithelia were separated and digested with trypsin (Gibco) for 15 min at 37 °C. Keratinocytes were seeded in high-calcium medium [Ca^2+^-free and Mg^2+^-free EMEM (BioWhittaker, Lonza, Switzerland) supplemented with 4% Chelex-treated (Bio-Rad, Hercules, CA) foetal bovine serum (Cultek, Madrid, Spain) and 0.2 mmol·L^−1^ Ca^2+^]. After overnight culture for cell attachment, the dishes were washed three times with cold PBS, and growth medium (CnT-24, Cellntec, Switzerland) was added. Then, the keratinocytes were allowed to grow to confluence. The keratinocytes were subjected to consecutive passages at low dilution using high-calcium medium for seeding. Cells that had undergone fewer than five passages were used for western blot analysis.

For proliferation studies, cells were incubated with 10 µmol·L^−1^ BrdU for 1 h, and BrdU incorporation was detected by immunohistochemistry. The detection of senescence was carried out by using a senescence β-galactosidase staining kit (9860, Cell Signaling). In colony-forming assays, growing colonies were fixed and stained with Coomassie blue, and the number of colonies was counted.

### Flow cytometry analysis of cell populations from oral epithelia

Epithelial keratinocytes and submucosal cells were harvested from the oral tissue (hard and soft palate) of 9-week-old K5-IKKβ and control mice and analysed as previously described for skin cells.^7^

### Protein extraction and western blot analysis

Total protein extracts (30 μg) from cultured mouse oral keratinocytes, tumours or tissues were prepared following standard techniques. The antibodies used are listed in Supplementary Table S1.

### RNA isolation and real-time PCR

Total RNA, including miRNA, was isolated from cell cultures from oral tissue by using the miRNeasy Mini Kit (Qiagen) according to the manufacturer’s instructions. The reverse transcription reaction was performed by using the High-Capacity cDNA Reverse Transcription Kit (Applied Biosystems) for mRNA and the TaqMan MicroRNA Reverse Transcription Kit (Applied Biosystems) for miRNA. Quantitative qRT-PCR was performed in a 7500 Fast Real-Time PCR System using GoTaq qPCR Master Mix (Promega) for mRNA and TaqMan Universal PCR Master Mix (Applied Biosystems) for miRNA. The sequences of the oligonucleotides used are provided in.^7^ For miRNA, TaqMan probes were used. *TBP* was used for the normalization of mRNA expression, and SnoRNA202 and SnoRNA234 were used for the normalization of miRNA expression.

### CT analysis

CT studies were performed in a small-animal Argus PET-CT scanner (SEDECAL, Madrid, Spain) in mice that were anaesthetized by the inhalation of 2%–2.5% isoflurane in 100% oxygen, with the following acquisition parameters: voltage 45 kV, current 150 μA, 8 shots, 360 projections and standard resolution.

Images were analysed using the image analysis software ITK-SNAP.

### Statistical analysis

For mRNA expression, BrdU incorporation, colony formation and SA-β-galactosidase assays, *p*-values were determined by using the unpaired, two-tailed Student’s *t*-test. For flow cytometry assays, the Mann-Whitney test was used. All experiments were performed at least three times. *P* < 0.05 were considered significant, and the data were expressed as the mean ± SEM. For miRNA expression, we employed REST 2009 Software (Qiagen), which calculates statistical significance using bootstrapping and randomization algorithms.^40^

## Supplementary information

Supplementary Figures S1-S3

Supplementary Table S1
