# Supplementary figures and images for: IKKβ overexpression together with a lack of tumour suppressor genes causes ameloblastic odontomas in mice
Source: Int J Oral Sci. 2020 Jan 2;12:1. doi: 10.1038/s41368-019-0067-9 (PMC6946653; doi:10.1038/s41368-019-0067-9)

## Supplementary Figure S1

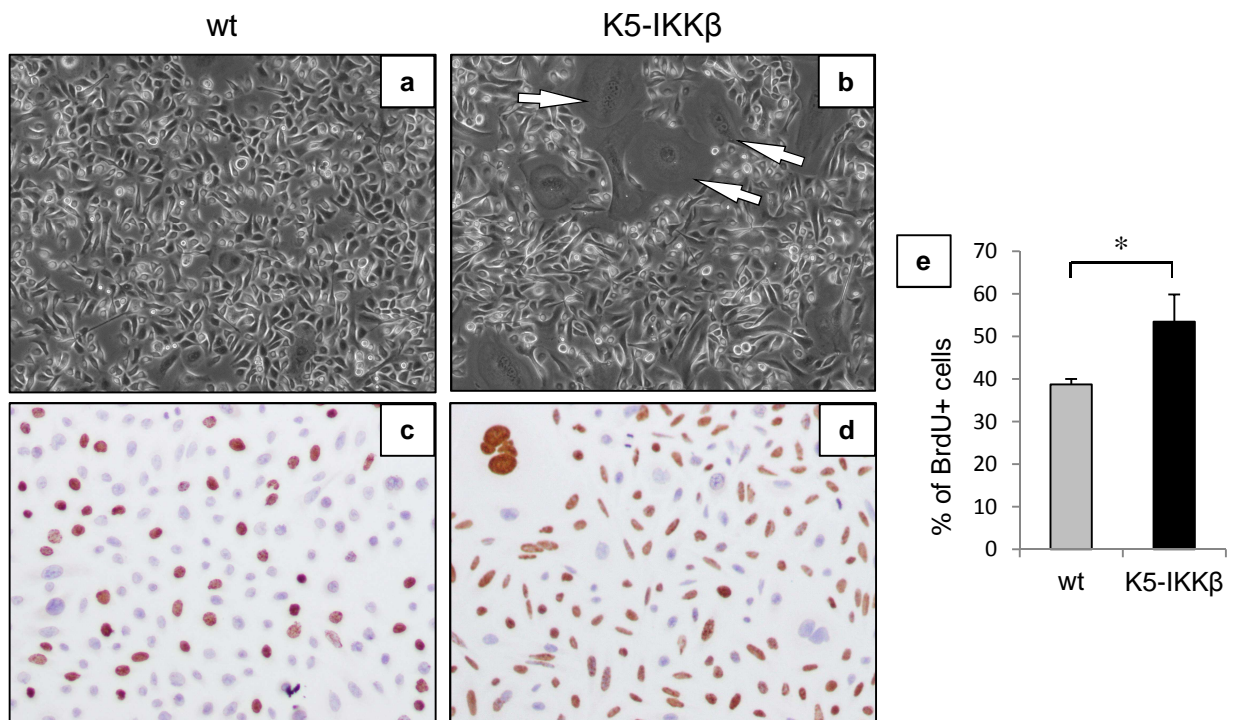

## Supplementary Figure S2

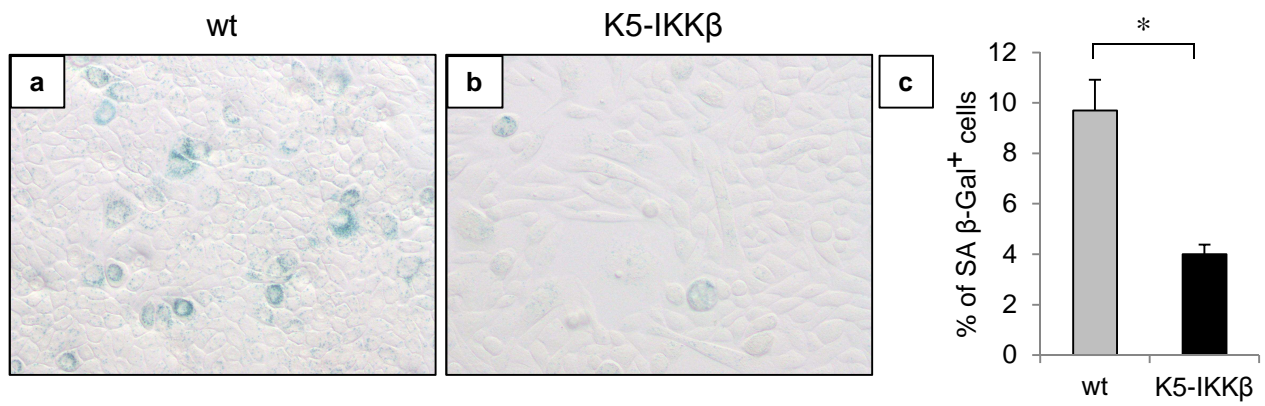

## Supplementary Figure S3

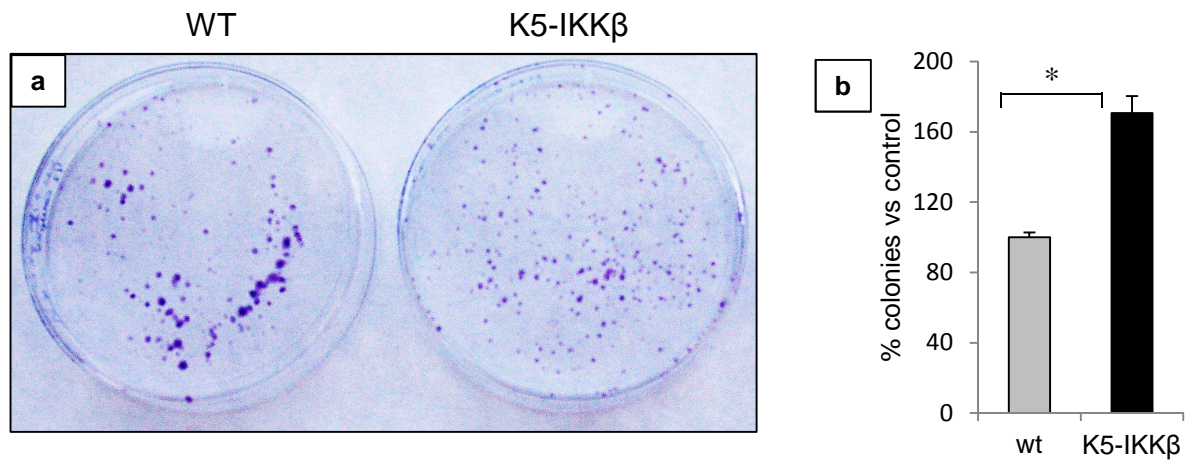

Supplement: Supplementary file 1 — Supplementary Figures S1-S3 [file 41368_2019_67_MOESM1_ESM.pdf]
