# Supplementary material for: IKKβ overexpression together with a lack of tumour suppressor genes causes ameloblastic odontomas in mice
Source: Int J Oral Sci. 2020 Jan 2;12:1. doi: 10.1038/s41368-019-0067-9 (PMC6946653; doi:10.1038/s41368-019-0067-9)
Supplement: Supplementary file 2 — Supplementary Table S1 [file 41368_2019_67_MOESM2_ESM.pdf]

Supplementary Table S1: list of antibodies used in this work

| Target | Clone | Reference | Source | Use |
|---|---|---|---|---|
| IKKβ | 10AG2 | NB100-56509 | Novus Biologicals | IHC, WB |
| Keratin K5 | Poly19055 | PRB-160P | Covance | IHC |
| Phospho-p65 (Ser536) | | 3031 | Cell Signaling Technology | IHC |
| Phospho-p65 (Ser536) | 93H1 | 3033 | Cell Signaling Technology | WB |
| p65 | D14E12 | 8242 | Cell Signaling Technology | WB |
| Phospho-IκBα (Ser32) | 14D4 | 2859 | Cell Signaling Technology | WB |
| IκBα | C21 | sc-371 | Santa Cruz Biotechnology | WB |
| p53 | CM5 | NCL-p53-CM5p | Novocastra, Leica Biosystems | WB |
| p19 | | ab80 | Abcam | IHC, WB |
| p16 | | sc1207 | Santa Cruz Biotechnology | WB |
| p63 | 4A4 | ab735 | Abcam | IHC |
| Vimentin | VI-01 | ab7752 | Abcam | IHC |
| β-catenin | | 610153 | BD Transduction Laboratories | IHC, WB |
| active β-catenin | 8E7 | 05-665 | Upstate Biotechnology | WB |
| Phospho-AKT (Ser743) | 376E11 | 3787 | Cell Signaling Technology | IHC |
| Phospho-AKT (Ser743) | D9E | 4060 | Cell Signaling Technology | WB |
| AKT | E45 | ab32038) | Abcam | WB |
| Phospho-STAT3 (Tyr705) | | 9131 | Cell Signaling Technology | WB |
| STAT3 | 79D7 | 4904 | Cell Signaling Technology | WB |
| MMP2 | | 4022 | Cell Signaling Technology | WB |
| MMP9 | | 19016 | Millipore | WB |
| GAPDH | 6C5 | sc32233 | Santa Cruz Biotechnology | WB |
| CD45 | | ab10558 | Abcam | IHC |
| biotin-CD45.2 | 104 | 553771 | BD Pharmingen | FC |
| CD11b | EPR1344 | ab133357 | Abcam | IHC |
| PE-CD11b | M1/70 | 561689 | BD Pharmingen | FC |
| PE-CD3e | 145-2C11 | 553064 | BD Pharmingen | FC |
| γδ-TCR | GL3 | 553175 | BD Pharmingen | FC |
| β-Actin | | sc-1616 | Santa Cruz Biotechnology | WB |
